# MicroRNA-140-5p ameliorates the high glucose-induced apoptosis and inflammation through suppressing TLR4/NF-κB signaling pathway in human renal tubular epithelial cells

**DOI:** 10.1042/BSR20192384

**Published:** 2020-03-04

**Authors:** Jie Su, Jian Ren, Haiyan Chen, Bo Liu

**Affiliations:** 1 Department of Endocrinology, Luoyang Central Hospital Affiliated to ZhengZhou University, Luoyang 471000, Henan, China; 2 Department of Endocrinology, Zhengzhou Central Hospital Affiliated to ZhengZhou University, Zhengzhou 450000, Henan, China; 3 Department of Neurosurgery, 150th Central Hospital of Chinese People’s Liberation Army, Luoyang 471000, Henan, China

**Keywords:** apoptosis, diabetic nephropathy, microRNA-140-5p, pro–inflammatory cytokines, renal tubular cell, TLR4/NF-κB pathway

## Abstract

Hyperglycemia-induced renal tubular cell injury is thought to play a critical role in the pathogenesis of diabetic nephropathy (DN). However, the role of miRNAs in renal tubular cell injury remains to be fully elucidated. The aim of the present study was to investigate the role and mechanisms of miRNAs protecting against high glucose (HG)-induced apoptosis and inflammation in renal tubular cells. First, we analyzed microRNA (miRNA) expression profiles in kidney tissues from DN patients using miRNA microarray. It was observed that miRNA-140-5p (miR-140-5p) was significantly down-regulated in kidney tissues from patients with DN. An inverse correlation between miR-140-5p expression levels with serum proteinuria was observed in DN patients, suggesting miR-140-5p may be involved in the progression of DN. HG-induced injury in HK-2 cells was used to explore the potential role of miR-140-5p in DN. We found that miR-140-5p overexpression improved HG-induced cell injury, as evidenced by the enhancement of cell viability, and inhibition of the activity of caspase-3 and reactive oxygen species (ROS) generation. It was also observed that up-regulation of miR-140-5p suppressed HG induced the expressions of pro-inflammatory cytokines, such as tumor necrosis factor-α (TNF-α), interleukin (IL)-1β and IL-6 in HK-2 cells. In addition, TLR4, one of the upstream molecules of NF-κB signaling pathway, was found to be a direct target of miR-140-5p in the HK-2. Moreover, the HG-induced activation of NF-κB signaling pathway was inhibited by miR-140-5p overexpression. These results indicated that miR-140-5p protected HK-2 cells against HG-induced injury through blocking the TLR4/NF-κB pathway, and miR-140-5p may be considered as a potential prognostic biomarker and therapeutic target in the treatment of DN.

## Introduction

Diabetic nephropathy (DN) is one of the most relevant microvascular complications of diabetes, which is the leading cause of end-stage renal disease throughout the world [1]. In China, DN has become a major public health problem, bringing huge social and economic burdens to individuals, families and society [2]. Although renal tubular cell injury has been considered as the most lethal attributes of DN, the mechanism underlying is still limited.

It is known that renal tubular cell injury is one of the main characteristics of DN and increasing evidences demonstrate the involvement of oxidative stress, inflammation and cell apoptosis during renal tubular cell injury [3]. Hyperglycemia triggers the generation of excessive reactive oxygen species (ROS), which leads to increased apoptosis of renal tubular epithelial cells, resulting in tubular injury [4,5]. Additionally, abnormal inflammation can directly destroy renal architecture, along with increased levels of inflammatory cytokines, such as interleukin (IL)-1β, tumor necrosis factor-α (TNF-α) and monocyte chemoattractant protein-1 (MCP-1) [6,7]. Therefore, therapeutic strategies that inhibit renal tubular epithelial cells apoptosis and inflammation are urgently required.

MicroRNAs (miRNAs) are ∼21 nucleotides, single-stranded, noncoding RNAs which induce mRNA cleavage, or translational repression by base-pairing with the 3′-UTR region of target mRNAs [8]. Cumulative studies have shown that miRNAs are involved in high glucose (HG)-mediated apoptosis in various types of cells. For example, inhibition of miR-183 prevented HG-elicited apoptosis by repressing peroxiredoxin 3 (PRDX3) expression in retinal pigment epithelial (RPE) cells [9]. Lin et al. [10] have shown that miR-199a-5p mediated HG-induced ROS production and apoptosis in pancreatic β-cells by targeting SIRT1. MiR-29c has been related to podocyte apoptosis by targeting Sprouty homolog 1 [11]. Notably, several studies have shown that various functions in renal tubular cell such as proliferation, cell adhesion and epithelial–mesenchymal transition (EMT) are finely regulated by miRNAs. For example, Tian et al. [12] have reported that up-regulated miR-485 suppressed apoptosis of renal tubular epithelial cells in mice via regulating the TGF-β-MAPK signaling pathway by inhibiting RhoA expression. However, few studies have been reported on whether miRNAs are involved in the regulation of hyperglycemia-induced renal tubular cell apoptosis and inflammation in DN disease.

In the current study, the miRNA expression profile was examined in kidney tissues from patients with DN. Furthermore, we established an HG-induced renal tubular cell injury model and investigated the role of miRNA-140-5p (miR-140-5p)/TLR4/NF-κB pathway in preventing HG-induced renal tubular cell injury.

## Materials and methods

### Tissue samples

Peripheral blood samples were obtained from 20 patients with DN and 20 healthy controls by standard protocol at Department of Neurosurgery, 150th Central Hospital of Chinese People’s Liberation Army from January 2017 to June 2018. The kidney tissues were from patients diagnosed with DN or kidney carcinoma without diabetes as the control. All patients with DN were diagnosed based on the presence of diabetes, massive proteinuria, and other histological changes typical of DN. All experimental protocols were approved by the Ethics Committee of the 150th Central Hospital of Chinese People’s Liberation Army. Informed consent was obtained from all patients.

### Cell culture and treatment

HK-2 cells were obtained from the American Type Culture Collection (ATCC, Manassas, VA, U.S.A.) and cultured in Dulbecco’s modified Eagle’s medium (DMEM, Thermo Fisher Scientific, Waltham, MA, U.S.A.) supplemented with 10% fetal bovine serum (FBS, PAN-Biotech, Aidenbach, Germany) and 1% penicillin–streptomycin (Beyotime, Jiangsu, China) in a 5% CO_2_ incubator at 37°C. When the cells reached 90% confluence, 5.5 mmol/l (Control group) or 30 mM (HG group) in serum-free DMEM was added to HK-2 cells.

### miRNA microarray

Total RNA was isolated from three peripheral blood samples from patients with DN and three peripheral blood samples from healthy controls by miRNeasy isolation kit (Qiagen, Milan, Italy) according to the manufacturer’s protocol. The RNA quantity was assessed by NanoDrop ND-1000 spectrophotometry (Thermo Fisher Scientific, Inc., Waltham, MA, U.S.A.). Total RNA (200 ng) was labeled using the miRCURY Hy3/Hy5 Power Labeling kit and hybridized on the miRCURY™ LNA array (v.16.0; Exiqon A/S, Copenhagen, Denmark) according to the manufacturer’s protocol. Scanned images from Axon GenePix 4000B microarray scanner (Axon Instruments, Foster City, CA, U.S.A.) were imported into the GenePix Pro6.0 program (Axon Instruments) for grid alignment and data extraction. The miRNAs with intensities ≥50 were used to calculate a normalization factor in all samples. The heatmap of the 50 miRNAs with the most marked differences was created using a method of hierarchical clustering by GeneSpring GX, version 7.3 (Agilent Technologies, Inc.).

### Reverse transcription-quantitative polymerase chain reaction analysis

Total RNA was extracted from peripheral blood samples, kidney samples or cells with the TRIzol reagent (Invitrogen, U.S.A.) according to the manufacturer’s protocol. For miRNA and mRNA reverse transcription, cDNA was synthesized using the miRNA reverse transcription kit (Applied Biosystems; Thermo Fisher Scientific, Inc.) and the PrimeScript reverse transcription reagent kit (Takara Biotechnology Co., Ltd., Dalian, China) according to the manufacturer’s instructions, respectively. Real-time PCR for miRNA and mRNA were performed using a standard protocol from the SYBR Green PCR kit (Toyobo, Osaka, Japan) on an ABI 7300 system (Applied Biosystems; Thermo Fisher Scientific, Inc.). Relative quantification was determined by normalization to U6 or GAPDH. The primers for qRT-PCR analysis were as follows: miR-140-5p forward: 5′-GAGTGTCAGTGGTTTTACCCT-3′; miR-140-5p reverse: 5′-GCAGGGTCCGAGGTATTC-3′; U6 forward: 5′-TGCGGGTGCTCGCTTCGCAGC-3′; U6 reverse: 5′-CCAGTGCAGGGTCCGAGGT-3′; TLR4 forward: 5′-CTGGGATGCCGTGTTATTT-3′, TLR4 reverse: 5′-TAGGAGGTGCGAGTTCAGGT-3′; GAPDH forward: 5′-AGGTCGGTGTGAACGGATTTG-3′, GAPDH reverse: 5′-TGTAGACCATGTAGTTGAGGTCA-3′. The thermocycling conditions were as follows: 50°C for 2 min, followed by 40 cycles of 95°C for 15 s and 60°C for 1 min. The qRT-PCR assays were performed in triplicate and the relative expression levels were calculated based on the 2^−ΔΔ*C*_t_^ method [13].

### Cell transfection

The miR-140-5p mimics, mimics negative control (mimics NC), miR-140-5p inhibitor, inhibitor negative control (inhibitor NC) and TLR4 overexpression plasmid were all provided by Shanghai GenePharma Co., Ltd. (Shanghai, China). When HK-2 cells in six-well plate grown to approximately 80% confluence, miR-140-5p mimics (25 nmol/l), miR-140-5p inhibitor (25 nmol/l) or 2 μg pcDNA-TLR4 were transfected into cells at 37°C for 24 h, using Lipofectamine® 2000 (Invitrogen). After 6 h transfection, the cells were stimulated with HG (30 mM) for 24 h and then protein and RNA were extracted for analyses.

### MTT assay

Cell viability of HK-2 was evaluated 24 h after exposure to the various treatments. Briefly, 20 μl of MTT solution (5 mg/ml, Sigma, St. Louis, MO, U.S.A.) was added to each well and incubated for 4 h. Absorbance was measured at 560 nm by a microplate reader (Bio-Tek Instruments, Germany).

### Flow cytometry assay

Apoptosis of HK-2 cells was evaluated 24 h after exposure to the various treatments. HK-2 cells (1 × 10^6^) were digested with trypsin, harvested and washed in ice-cold PBS, and stained in 500 µl binding buffer with 5 µl PI and 5 µl AnnexinV-FITC (Nanjing KeyGen Biotech Co. Ltd., Nanjing, China) for 20 min in the dark. The fluorescence signals were collected by EPICS XL-MCL FACScan (BectoneDickinson, Mountain View, CA, United States) and then analyzed by FlowJo 8.7.1 software (Ashland, OR).

### Caspase-3 activity assay

The caspase-3 activity in cell lysates was determined after exposure to the various treatments using a Caspase-3 Activity Assay kit (Beyotime Institute of Biotechnology), according to the manufacturer’s protocol.

### Measurement of ROS

ROS production in chondrocytes was measured using 2′,7′-DCF diacetate (DCFH-DA; Sigma–Aldrich; EMD Millipore) as previously reported [14]. After exposure to the various treatments, HK-2 cells in six-well plate were stained with 20 µmol/l DCFH for 30 min at 37°C. After that, cell nucleus was stained using DAPI staining. Cells were captured using a fluorescence microscope (IX-81; Olympus Corp., Shinjuku, Tokyo, Japan). The fluorescence intensity was analyzed by using the fluorescence plate reader (Titertek Plus MS 212, ICN, Eschwege, Germany) at Ex/Em = 488/525 nm. Fold-increases in ROS levels were determined by comparison with the control group.

### Detection of malonaldehyde, superoxide dismutase and glutathione peroxidase

After designated treatment, the HK-2 were lysed using lysis buffer (Beyotime Biotechnology), the lysates were collected for the detection of superoxide dismutase (SOD) and malondialdehyde (MDA) and glutathione peroxidase (GPx). Then, the kits of SOD (cat no.S0103), MDA (cat no.S0131) and GPx (cat no.S0056) (Beyotime Biotechnology, Shanghai, China) were used to examine the level of SOD, the content of MDA and the activity of GPx according to the manufacturer’s instructions.

### ELISA

After exposure to the various treatments, the supernatant was carefully collected by centrifugation at 2000 rpm for 20 min. The concentrations of IL-6, IL-1β and TNF-α levels were analyzed by using IL-6 (cat no. p1330), IL-8 (cat no. p1640) and TNF-α (cat no. pt518) ELISA kits according to the kit instructions. All ELISA kits were purchased from Beyotime Biotechnology, Shanghai, China.

### Luciferase assays

The 3′-UTR of TLR4, with wild-type (wt) or mutant (mut) binding sites for miR-140-5p, was amplified and cloned into the pGL3 vector (Promega Corporation, Madison, WI, U.S.A.) to generate the pGL3-wt-TLR4-3′-UTR plasmid or pGL3-Mut-TLR4-3′-UTR plasmid, respectively. For the luciferase reporter assay, HK-2 cells were co-transfected with the luciferase reporter vectors and miR-140-5p mimics, miR-140-5p inhibitor or corresponding negative control using Lipofectamine 2000 reagent. The pRL-TK plasmid (Promega Corporation) was used as a normalizing control. After 48 h, luciferase activity was analyzed using the Dual-Luciferase Reporter Assay system (Promega Corporation) according to the manufacturer’s protocol.

### NF-κB activity assay

HK-2 cells (1 × 10^6^ cells/well) were plated in six-well tissue culture plates for 24 h. Then, cells were transfected with 2.5 μg of an NF-κB reporter luciferase construct. Six hours later, cells were washed and then treated with miR-140-5p mimics for another 24 h, followed by 10 ng/ml HG for 24 h. Cells were then washed in PBS and harvested in 500 μl of 1× passive lysis buffer. Luciferase was quantified using Promega luciferase assay kit on a luminometer. Experimental values were recorded relative to untreated control samples.

### Western blot

Total protein was extracted using RIPA lysis buffer (Beyotime Biotechnology, Shanghai, China) supplemented with protease inhibitors (Roche, Guangzhou, China). The extraction and isolation of nuclear and cytoplasmic proteins were performed according to the Nuclear and Cytoplasmic Protein Extraction Kit (Beyotime Biotechnology, Shanghai, China). The nuclear and cytoplasmic proteins were quantified using a BCA kit (Beyotime Institute of Biotechnology, Haimen, China). Next, the proteins in the lysates were separated on SDS/PAGE gels and electrotransferred to PVDF membranes (GE Healthcare, Freiburg, DE), followed by blocking in 5% skim milk solution for 1 h at room temperature. Primary antibodies against TLR4 (cat. no. 14358; 1:2000), nuclear p-p65 (cat no. #3033, Cell Signaling Technology, 1:1000 dilution), phosphorylated IκB-α (p-IκB-α; cat no. #2859, Cell Signaling Technology, 1:1000 dilution), IκB-α (cat no.sc-52900, Santa Cruz Biotechnology, 1:1000 dilution), Histone H3 (cat no. #9728, 1:2000, Cell Signaling Technology, 1:1000 dilution) and β-actin (cat no.#4970, Cell Signaling Technology, 1:2000 dilution) were incubated at 4°C overnight. Then, membranes were incubated with the corresponding horseradish peroxidase–conjugated goat anti-rabbit or anti-rat secondary antibodies (cat no. ab6721 and 6785, 1:2000, Abcam, Cambridge, U.K.) for 1 h at room temperature. The protein bands were visualized using ECL detection reagent (GE Healthcare Life Sciences, Piscataway, NJ, U.S.A.). The intensity of protein fragments was quantified with Bio-Rad Laboratories Quantity One software 3.0 (Bio-Rad Laboratories, Inc.). β-actin was used as the inner control of the cytoplasmic proteins; Histone H3 was used as the inner control of the nuclear proteins.

### Statistical analysis

Data were presented as mean ± S.D. GraphPad Prism 5 software (GraphPad Software, Inc., La Jolla, CA, U.S.A.) was used to perform all the statistical analyses. One-way analysis of variance followed by Tukey’s post-hoc test was applied to compare differences between multiple groups. For correlation of miR-140-5p and TLR4 expression, the data were analyzed using Spearman’s correlation. *P*≤0.05 was considered as statistically significant.

## Results

### miR-140-5p was down-regulated in kidney tissues from patients with DN

**Figure 1:**
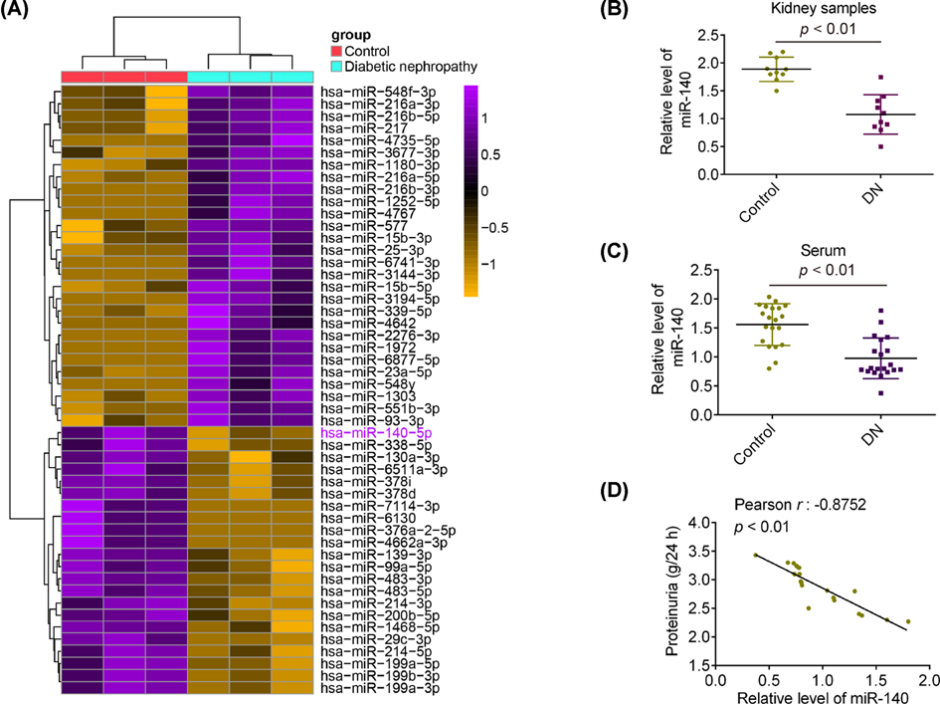
miR-140-5p is down-regulated in kidney tissues and peripheral blood from patients with DN (**A**) Heatmap of normalized expression levels of miRNAs in peripheral blood from patients with DN and healthy controls (*n*=3/group). Green indicates low expression levels; red indicates high expression levels. (**B**) Expression of miR-140-5p was detected by qRT-PCR analysis in ten kidney tissues from patients with DN and ten kidney tissues from healthy controls. *P*<0.01, vs. control group. (**C**) Expression of miR-140-5p was determined by qRT-PCR analysis in 20 peripheral blood samples from patients with DN and 20 peripheral blood samples from healthy controls. *P*<0.01, vs. control group. (**D**) Pearson correlation analysis demonstrated the inverse correlations of plasma miR-140-5p with proteinuria in 20 DN patients (r: −0.8752, *P*<0.01).

First, microarray was performed to compare the miRNA patterns in kidney tissues from DN patients and healthy controls. The data identified 50 differentially expressed miRNAs (21 were up-regulated and 29 were down-regulated) (Figure 1A). Among the aberrantly expressed miRNAs, miR-140-5p was selected for further investigation as its expression level was one of the lowest miRNAs in the DN patient group. Consistent with our results, miR-140-5p was also found to be down-regulated in kidney tissues from patients with DN [15], suggesting that miR-140-5p may be involved in the progression of DN. In addition, several studies have shown that miR-140-5p improved many types of cell injured models through regulation of inflammation and apoptosis [16–21]. However, whether miR-140-5p has a protective effect against hyperglycemia-induced tubular cell injury in DN remains to be elucidated. Thus, we selected miR-140-5p for further investigation.

To further validate the expression pattern of miR-140-5p obtained from miRNA microarray assay, qRT-PCR was performed to detect miR-140-5p in ten kidney tissues from patients with DN and ten kidney tissues from healthy controls by qRT-PCR. Compared with the control group, miR-140-5p was significantly down-regulated in the kidney tissues from the patients with DN (Figure 1B). It was also observed that the expression of miR-140-5p was markedly down-regulated in peripheral blood samples from the patients with DN, compared with that in the control group, suggesting that miR-140-5p may serve as a promising diagnostic marker for DN (Figure 1C). More importantly, an inverse correlation between miR-140-5p levels and proteinuria was also observed in patients with DN (Figure 1D). All data indicate that miR-140-5p may be involved in the pathogenesis of DN.

### miR-140-5p is down-regulated in an HG-induced injury model of HK-2

**Figure 2:**
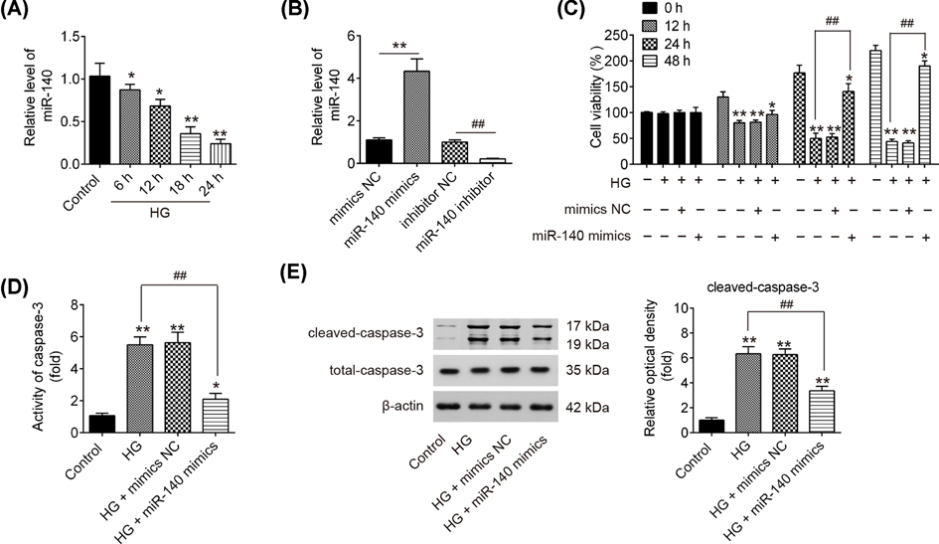
Overexpression of miR-140-5p inhibits HG-induced HK-2 cell apoptosis HK-2 cells were transfected with miR-140-5p mimics for 24 h, followed by treatment with 30 mM HG, and then cells were harvested for subsequent experiments. (**A**) Expression of miR-140-5p was determined by qRT-PCR analysis at different times after HG treatment. (**B**) Expression of miR-140-5p was determined by qRT-PCR analysis after miR-140-5p mimics and miR-140-5p inhibitor transfection. (**C**) Cell viability was assessed using MTT assay at 0, 12, 24 and 48 h, respectively. (**D**) Activity of caspase-3 was measured using a commercial kit. (**E**) The expression of cleaved caspase-3 was measured by Western blot. Data were represented as the mean ± SD of three independent experiments. *P<0.05, **P<0.01, vs. control group; ^##^*P*<0.01, vs. HG group.

To further examine the role of miR-140-5p in DN, we constructed an *in vitro* model through HG treatment in HK-2 cells, which is widely used for the research of DN [22]. Following treatment of the HK-2 cells with 30 mM HG at different times, the expression of miR-140-5p declined in a dose-dependent manner compared with control group, which was consistent with the results in the clinical samples (Figure 2A). Subsequently, we transfected miR-140-5p mimics and miR-140-5p inhibitor into the cultured HK-2 cells and examined the effects on cell viability and apoptosis. The results showed that miR-140-5p was markedly increased (decreased) after miR-140-5p mimics (miR-140-5p inhibitor) transfection in HK-2 cells (Figure 2B). Subsequently, the cell viability, caspase-3 activity, and the expression of apoptosis-associated proteins were evaluated. MMT assay showed that the cell viability was gradually decreased after HG stimulation at different times, while overexpression of miR-140-5p significantly attenuated the inhibitory effects of HG on the viability of HK-2 cells (Figure 2C). Moreover, we confirmed that HG obviously elevated the activity of caspase-3 and the level of cleaved-caspase-3, compared with the control group in HK-2 cells, however, HG-induced elevation was significantly inhibited by overexpression of miR-140-5p (Figure 2D,E). Taken together, these data indicated that the overexpression of miR-140-5p protected the HK-2 cells against HG-induced apoptosis, suggesting that miR-140-5p may be a key protective factor in DN.

### Overexpression of miR-140-5p attenuated the ROS generation in HG-induced HK-2 cells

**Figure 3:**
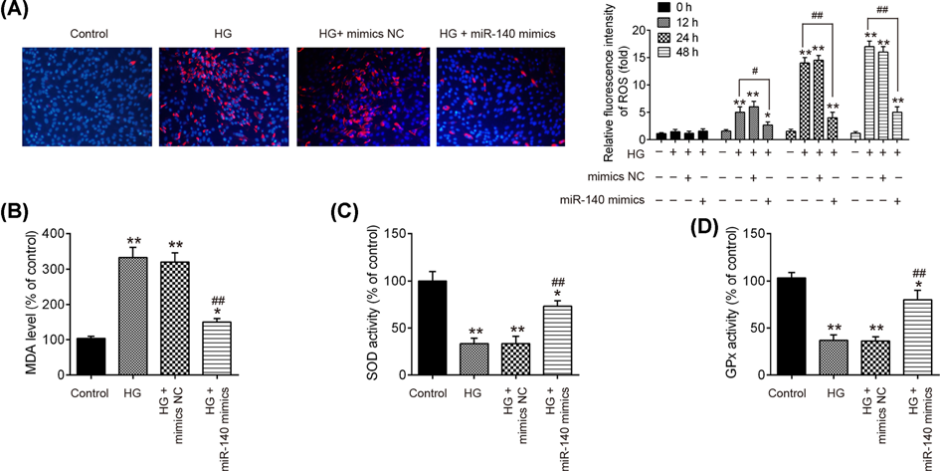
Overexpression of miR-140-5p attenuated the ROS generation in HG-induced HK-2 cells HK-2 cells were transfected with miR-140-5p mimics for 24 h, followed by treatment with 30 mM HG, and harvesting of cells for subsequent experiments. (**A**) Production of ROS was detected using DCFH-DA and DAPI staining (magnification ×200) at 0, 12, 24 and 48 h, respectively. The image was captured at 48 h after HG stimulation. (**B**–**D**) The expression levels of MDA and the activity of SOD and GPx were measured by MDA, SOD and GPx assay kits at 24 h after HG stimulation. Data were represented as the mean ± SD of three independent experiments. *P<0.05, **P<0.01, vs. control group; ^##^*P*<0.01, vs. HG group.

It was suggested that ROS contributes to tubular injury via the induction of renal tubular epithelial cells apoptosis in DN [23]. Thus, inhibition of ROS may be an effective therapeutic strategy for protection against HG-induced tubular injury [24]. Thus, to determine whether miR-140-5p attenuated the HG-induced renal tubular injury through suppression of the ROS generation, DCFH-DA assay was used to analyze ROS production of HK-2 cells. The results indicated that HG gradually increased the ROS production time-dependently, while overexpression of miR-140-5p significantly decreased ROS production caused by HG in HK-2 cells (Figure 3A). In addition, the commonly used biomarkers of oxidative stress including MDA, SOD and GPx [25] were employed for the assessments of miR-140-5p on oxidative stress. As show in Figure 3B–D, the content of MDA was significantly increased, but the activities of SOD and GPx were notably decreased after HG stimulation compared with that in control group. However, overexpression of miR-140-5p abolished HG-induced oxidative stress in HK-2 cells. These data indicated that miR-140-5p up-regulation protected the HK-2 against HG-induced ROS production.

### Overexpression of miR-140-5p attenuated the HG-induced inflammatory response in HK-2 cells

**Figure 4:**
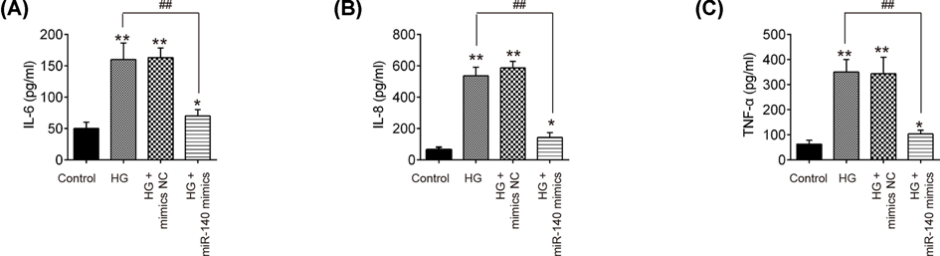
Overexpression of miR-140-5p attenuated the HG-induced inflammatory response in HK-2 cells HK-2 cells were transfected with miR-140-5p mimics for 24 h, followed by treatment with 30 mM HG for 24 h, and harvesting of cells for subsequent experiments. (**A**–**C**) The levels of IL-6, IL-8 and TNF-α were measured by ELISA kits. Data were represented as the mean ± SD of three independent experiments. *P<0.05, **P<0.01, vs. control group; ^##^*P*<0.01, vs. HG group.

Increasing evidence supports that inflammation is engaged in the DN pathogenesis [26]. Therefore, we also investigated the effect of miR-140-5p up-regulation on the HG-induced inflammatory response in HK-2 cells. As shown in Figure 4A–C, the levels of pro-inflammatory cytokines, such as IL-6, IL-8 and TNF-α were significantly increased after HG stimulation, whereas the increased pro-inflammatory cytokines induced by HG was markedly reduced by overexpression of miR-140-5p. These data suggest that miR-140-5p up-regulation inhibited HG-induced inflammatory response in HK-2 cells.

### TLR4 was a direct target of miR-140-5p in HK-2 cells

**Figure 5:**
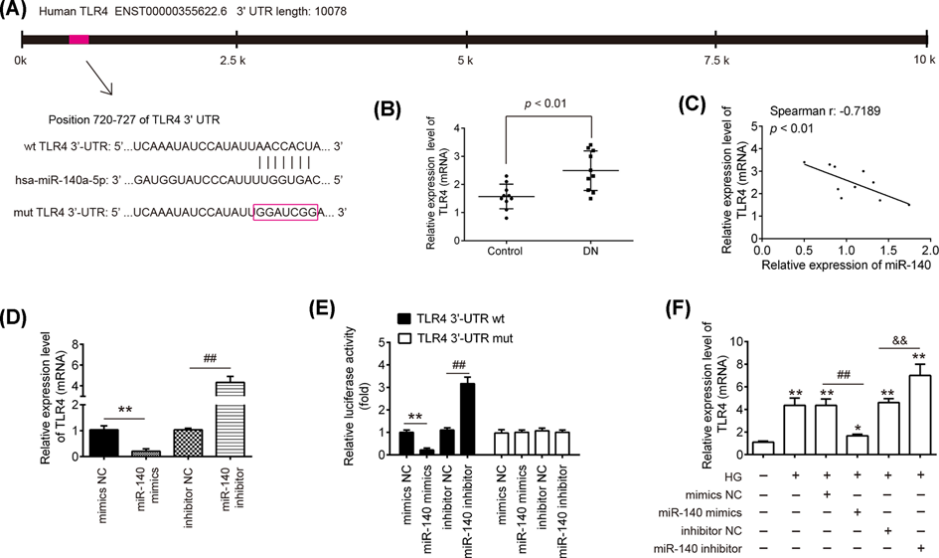
TLR4 was a direct target of miR-140-5p in HK-2 cells (**A**) The putative binding site of miR-140-5p and TLR4 is shown. (**B**) qRT-PCR analysis was used to assess the expression levels of TLR4 in ten kidney tissues from patients with DN and ten kidney tissues from healthy controls. *P*<0.01, vs. control group. (**C**) Spearman’s correlation was used to assess the correlation of miR-140-5p and TLR4 expression in kidney tissues from DN patients (r: −0.8183; *P*<0.01). (**D**) HK-2 cells were transfected with miR-140-5p mimics and miR-140-5p inhibitor for 24 h, and the cells were harvested. Then, the protein levels of TLR4 were analyzed by Western blot. (**E**) HK-2 cells were co-transfected with firefly luciferase constructs containing the TLR4 wild-type or mutated 3′-UTRs and miR-140-5p mimics, mimics NC, miR-140-5p inhibitor or inhibitor NC, and then the luciferase activity was analyzed using the dual-luciferase reporter assay system. Data represent the mean ± SD of three independent experiments. **P<0.01 vs mimics NC; ^##^*P*<0.01 vs inhibtor NC. (**F**) HK-2 cells were transfected with miR-140-5p mimics, mimics NC, miR-140-5p inhibitor or inhibitor NC for 24 h and then exposed to HG for 24 h, the expression of TLR4 was measured by qRT-PCR. Data represent the mean ± SD of three independent experiments. *P<0.05, **P<0.01 vs control group; ^##^*P*<0.01 vs HG+ mimics NC; ^&&^*P*<0.01 vs HG + inhibitor NC.

Considering the protective roles of miR-140-5p in HG-induced HK-2 cell injury, it may be beneficial to identify the downstream targets of miR-140-5p. Using TargetScan 7.0 (targetscan.org/) and miRanda (microrna.org/). TLR4, one of the upstream molecules of NF-κB signaling pathway, was identified as a potential target of miR-140-5p (Figure 5A). We also measured the expression of TLR4 in kidney tissues from ten patients with DN and ten healthy controls. As shown in Figure 5B, TLR4 was significantly up-regulated in the kidney tissues from the patients with DN, compared with that in the control group. Moreover, an inverse relationship between miR-140-5p expression levels and TLR4 in kidney tissues was observed (Figure 5C). To further identify whether the TLR4 levels was regulated by miR-140-5p, the effect of miR-140-5p mimics and inhibitor on the protein levels of TLR4 in HK-2 cells were examined by Western blot. As shown in Figure 5D, the protein expression of TLR4 was markedly down-regulated after miR-140-5p mimics transfection, but up-regulated by miR-140-5p inhibitor transfection. To experimentally validate whether TLR4 was a direct target of miR-140-5p, a luciferase reporter assay was performed. As shown in Figure 5E, the luciferase activity of the TLR4-3′UTR wt reporter plasmid was significantly reduced in HK-2 cells transfected miR-140-5p mimics, but increased in HK-2 cells transfected miR-140-5p inhibitor, compared with NC group. However, the luciferase activity of TLR4-3´UTR mut reporter plasmid showed no significant change (Figure 5E), suggesting that miR-140-5p regulated TLR4 expression by binding TLR4-3′UTR. These results demonstrated that TLR4 is a direct target of miR-140-5p. In addition, in the HG-treated HK-2 cells, the results also showed that the mRNA level of TLR4 was markedly up-regulated after HG-stimulation and overexpression of miR-140-5p attenuated the promoting effect of HG on the expression of TLR4, while miR-140-5p inhibitor enhanced this effect of HG (Figure 5F). These results indicated that miR-140-5p may inhibit renal tubular epithelial cell apoptosis and inflammatory response by targeting TLR4 in HG-induced cell injury model.

### miR-140-5p blocked the HG-induced activation of NF-κB pathway

**Figure 6:**
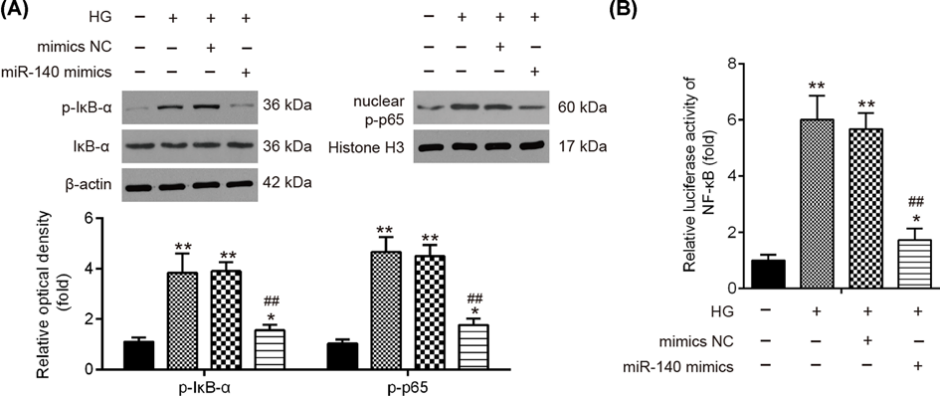
miR-140-5p blocked the HG induced activation of NF-κB pathway HK-2 cells were transfected with miR-140-5p mimics for 24 h, and then protein was extracted at 24 h after HG stimulation. (**A**) Levels of IκBα, p-IκB-α and nuclear p-p65 were assessed by performing Western blotting. The bands were semi-quantitatively analyzed by using ImageJ software, normalized to β-actin or Histone H3 density. (**B**) Relative luciferase activity of NF-κB was quantified using Promega luciferase assay kit on a luminometer. Data were represented as the mean ± SD of three independent experiments. *P<0.05, **P<0.01 vs. Control group; ^##^*P*<0.01 vs. HG + mimics group.

Since TLR4 is one of the upstream molecules of NF-κB signaling pathway, which is associated with the inflammatory response [27,28], further experiments were designed to examine whether miR-140-5p affects the activation of NF-κB pathway in HG-treated HK-2 cells. Levels of IκBα, p-IκB-α and nuclear p-p65 were assessed by performing Western blot. The results showed that the level of p-IκB-α and nuclear p-p65 was obviously increased after HG stimulation (Figure 6A). However, the increased expressions of p-IκB-α and nuclear p-p65 were markedly reduced by miR-140-5p overexpression in HK-2 cells. In addition, it was also found that the NF-κB activity was markedly increased after HG stimulation compared with control group, whereas this promoting effect was attenuated by miR-140-5p overexpression (Figure 6B). All data indicate that miR-140-5p may prevent HG-induced cell injury through blocking the activation of NF-κB pathway.

## Discussion

In the present study, we found that miR-140-5p was significantly down-regulated in kidney tissues and the peripheral blood of patients with DN, and miR-140-5p level was inversely correlated with proteinuria. Using an HG-induced HK-2 cell injury model, miR-140-5p overexpression inhibited HG-induced apoptosis, ROS generation, as well as inflammatory response through inhibition of the TLR4/NF-κB signaling pathway. These data indicated that miR-140-5p/TLR4/NF-κB axis may be an effective therapeutic target for DN patients.

Increasing evidences have demonstrated that miRNAs play a key role in the pathogenesis of DN [29,30]. For example, Wang et al. [31] found that miR-424 was down-regulated in renal tissues of DN rats and up-regulation of miR-424 obviously improved the symptoms in DN rat models by targeting Rictor. Yu et al. [32] showed that miR-370 was overexpressed in a rat model of DN and up-regulation of miR-370 promoted mesangial cell proliferation and extracellular matrix (ECM) by suppressing CNPY1 in DN rat models. Yao et al. [33] demonstrated that miR-874 was markedly down-regulated and miR-874 overexpression dramatically attenuated the inflammatory response in DN through targeting TLR4 in rats. These findings suggest that miRNAs maybe a potential therapeutic target for DN treatment. In our study, using an miRNA microarray assay, we found that miR-140-5p were significantly down-regulated in kidney tissues of patients with DN. Moreover, miR-140-5p levels were inversely correlated with proteinuria levels in DN patients. These results imply the potential role of miR-140-5p in human DN.

Multiple factors have been implicated in the progression of DN, but inflammatory cytokines and apoptosis in renal tubular cells may be critical in this disease [6,34,35]. Hyperglycemia leads to increased inflammatory cytokines of renal tubular epithelial cells and these inflammatory cytokines, such as IL-1, IL-6, IL-18, and TNF-α can directly damage renal tubular cells [36]. In addition, the influence of glucose-induced ROS production in the apoptosis of tubular cells has been described, which finally lead to DN progression [37]. Notably, several studies have shown that miRNAs are implicated in the inflammatory response and apoptosis of renal tubular cells. For example, Huang et al. [22] found that inhibition of miR-125b improved HG-induced HK-2 cell injury through blocking ROS formation and apoptosis. Yang et al. [38] showed that miR-374a suppressed the inflammatory response in HK2 cells through negative regulation of MCP-1 expression. However, whether miR-140-5p has anti-inflammatory and anti-apoptotic activities in DN remains unknown.

It has previously been reported that miR-140-5p is involved in cellular anti-inflammatory and anti-apoptotic processes in various types of cell models [39]. For example, Yang et al. [18] have found that miR-140-5p overexpression reduced the levels of inflammation in the *in vitro* model of acute lung injury (ALI) via blocking the TLR4/MyD88/NF-κB signaling pathway. Another study reported the inhibitory effects of miR-140-5p on cellular oxidative stress in acute kidney injury (AKI) by activating Nrf2/ARE pathway [40,41]. Based on the above researches, we hypothesized that miR-140-5p may affect the progression of DN through the regulation of renal tubular cell apoptosis, oxidative stress and inflammatory response. In the present study, using HG-induced injury model, we found that treatment of HK-2 cells with HG significantly decreased the expression of miR-140-5p in a time-dependent manner, which provided support for the possible role of miR-140-5p in HG-induced cell injury. Moreover, it was observed that up-regulation of miR-140-5p suppressed HG-induced apoptosis, ROS generation and inflammatory response, suggesting the protection role of miR-140-5p against HG-induced cell injury.

TLR4, an important regulator of the NF-κB signaling pathway, has been shown to regulate cell apoptosis, inflammatory response and oxidative damage of renal tubular epithelial cell in hyperglycemia [42,43]. Notably, a previous study showed that miR-140-5p inhibits pulmonary inflammatory response in ALI by targeting TLR4 [18]. However, whether TLR4 mediated the inhibitory effects of miR-140-5p on renal tubular cell apoptosis, oxidative stress and inflammatory response remained to be elucidated. In the present study, TLR4 was confirmed as a direct target gene of miR-140-5p in HK-2 cells. TLR4 is an important regulator of the NF-κB signaling pathway and the latter has been implicated in DN in recent years [44,45]. For example, Wang et al. [46] showed that umbelliferae (Umb) improved renal function through suppressing inflammatory response by blocking the activation of NF-κB pathway in a streptozotocin (STZ)-induced DN rat model. Zhu et al. [47] reported that Berberine ameliorated DN through relieving STZ-induced renal injury, inflammatory response via inactivating TLR4/NF-κB pathway in rats. To determine whether miR-140-5p suppressed the NF-κB signaling pathway by targeting TLR4 in HG-induced HK-2 injury model, the effects of miR-140-5p on the expressions of key kinases in NF-κB pathway were examined. The data showed that the NF-κB signaling pathway was induced in HG-treated HK-2 cells, as assessment by the expressions of key kinases in NF-κB pathway, and the overexpression of miR-140-5p blocked the activity of this pathway induced by HG, suggesting that the protective effect of miR-140-5p on HG-induced HK-2 cell injury may be mediated by the TLR4/NF-κB signaling pathway.

However, there are still some limitations in the present study. For example, multiple sources of ROS, including mitochondria, NADPH oxidases and NOS uncoupling, contribute to DN [48–50]. Interestingly, some reports indicate that ROS produced by mitochondria play a critical role in hyperglycemia-induced diabetic vascular complications [51,52]. In this study, we only detected global cellular ROS but not mitochondrial ROS. In future studies, we will distinguish the specific mechanism of ROS production. Moreover, although we elucidated the role and mechanisms of miR-140 protecting against HG-induced injury in renal tubular cells and obtained partial data from kidney tissues of DN patients, we did not further validate its therapeutic effects in mouse models. In future, we will further confirm if elevating miR140-5p in DN mice *in vivo* would have a therapeutic effect against DN.

In conclusion, we found overexpression of miR-140-5p attenuated HG-induced renal tubular epithelial cell injury by blocking the TLR4/NF-κB signaling pathway. These findings indicate that enhancing the expression of miR-140-5p may be a potential therapeutic approach for the treatment of DN.

## Abbreviations

ALI: acute lung injury
DCFH-DA: 2′,7′-DCF diacetate
DMEM: Dulbecco’s modified Eagle’s medium
DN: diabetic nephropathy
GPx: glutathione peroxidase
HG: high glucose
IL: interleukin
MCP-1: monocyte chemoattractant protein-1
MDA: malondialdehyde
miRNA: microRNA
miR-140-5p: miRNA-140-5p
p-IκB-α: phosphorylated IκB-α
ROS: reactive oxygen species
SOD: superoxide dismutase
STZ: streptozotocin
TNF-α: tumor necrosis factor-α

## References

[1] Liu Y. and Tang S.C. (2016) Recent progress in stem cell therapy for diabetic nephropathy. Kidney Dis. 2, 20–27 10.1159/000441913 27536688 PMC4946257

[2] Li H., Oldenburg B., Chamberlain C., O’Neil A., Xue B., Jolley D. et al. (2012) Diabetes prevalence and determinants in adults in China mainland from 2000 to 2010: a systematic review. Diabetes Res. Clin. Pract. 98, 226–235 10.1016/j.diabres.2012.05.010 22658670

[3] Habib S.L. (2013) Diabetes and renal tubular cell apoptosis. World J. Diabetes 4, 27–30 10.4239/wjd.v4.i2.27 23593533 PMC3627416

[4] Verzola D., Bertolotto M.B., Villaggio B., Ottonello L., Dallegri F., Salvatore F. et al. (2004) Oxidative stress mediates apoptotic changes induced by hyperglycemia in human tubular kidney cells. J. Am. Soc. Nephrol. 15, S85–S87 10.1097/01.ASN.0000093370.20008.BC 14684680

[5] Yu T., Sheu S.S., Robotham J.L. and Yoon Y. (2008) Mitochondrial fission mediates high glucose-induced cell death through elevated production of reactive oxygen species. Cardiovasc. Res. 79, 341–351 10.1093/cvr/cvn104 18440987 PMC2646899

[6] Rivero A., Mora C., Muros M., Garcia J., Herrera H. and Navarro-Gonzalez J.F. (2009) Pathogenic perspectives for the role of inflammation in diabetic nephropathy. Clin. Sci. 116, 479–492 10.1042/CS20080394 19200057

[7] Navarro-Gonzalez J.F., Mora-Fernandez C., Muros de Fuentes M. and Garcia-Perez J. (2011) Inflammatory molecules and pathways in the pathogenesis of diabetic nephropathy. Nat. Rev. Nephrol. 7, 327–340 10.1038/nrneph.2011.51 21537349

[8] Li T. and Cho W.C. (2012) MicroRNAs: mechanisms, functions and progress. Genomics Proteomics Bioinformatics 10, 237–238 23200132 10.1016/j.gpb.2012.10.002 PMC5054209

[9] Jiang Y., Sang Y. and Qiu Q. (2017) microRNA-383 mediates high glucose-induced oxidative stress and apoptosis in retinal pigment epithelial cells by repressing peroxiredoxin 3. Am. J. Transl. Res. 9, 2374–2383 28559987 PMC5446519

[10] Lin N., Li X.Y., Zhang H.M., Yang Z. and Su Q. (2017) microRNA-199a-5p mediates high glucose-induced reactive oxygen species production and apoptosis in INS-1 pancreatic beta-cells by targeting SIRT1. Eur. Rev. Med. Pharmacol. Sci. 21, 1091–1098 28338182

[11] Long J., Wang Y., Wang W., Chang B.H. and Danesh F.R. (2011) MicroRNA-29c is a signature microRNA under high glucose conditions that targets Sprouty homolog 1, and its in vivo knockdown prevents progression of diabetic nephropathy. J. Biol. Chem. 286, 11837–11848 10.1074/jbc.M110.194969 21310958 PMC3064234

[12] Tian Y., Han Y.X., Guo H.F., Jin H.T., Sun C., Qi X. et al. (2018) Upregulated microRNA-485 suppresses apoptosis of renal tubular epithelial cells in mice with lupus nephritis via regulating the TGF-beta-MAPK signaling pathway by inhibiting RhoA expression. J. Cell. Biochem. 119, 9154–9167 10.1002/jcb.27178 30145800

[13] Livak K.J. and Schmittgen T.D. (2001) Analysis of relative gene expression data using real-time quantitative PCR and the 2(-Delta Delta C(T)) Method. Methods 25, 402–408 10.1006/meth.2001.1262 11846609

[14] Tang Y., Vater C., Jacobi A., Liebers C., Zou X. and Stiehler M. (2014) Salidroside exerts angiogenic and cytoprotective effects on human bone marrow-derived endothelial progenitor cells via Akt/mTOR/p70S6K and MAPK signalling pathways. Br. J. Pharmacol. 171, 2440–2456 10.1111/bph.12611 24471788 PMC3997282

[15] Rudnicki M., Perco P., D Haene B., Leierer J., Heinzel A., Muhlberger I. et al. (2016) Renal microRNA- and RNA-profiles in progressive chronic kidney disease. Eur. J. Clin. Invest. 46, 213–226 10.1111/eci.12585 26707063

[16] Yang S., Li H. and Chen L. (2019) MicroRNA-140 attenuates myocardial ischemia-reperfusion injury through suppressing mitochondria-mediated apoptosis by targeting YES1. J. Cell. Biochem. 120, 3813–3821 10.1002/jcb.27663 30259997

[17] Han X.R., Wen X., Wang Y.J., Wang S., Shen M., Zhang Z.F. et al. (2018) MicroRNA-140-5p elevates cerebral protection of dexmedetomidine against hypoxic-ischaemic brain damage via the Wnt/beta-catenin signalling pathway. J. Cell. Mol. Med. 22, 3167–3182 10.1111/jcmm.13597 29536658 PMC5980153

[18] Yang Y., Liu D., Xi Y., Li J., Liu B. and Li J. (2018) Upregulation of miRNA-140-5p inhibits inflammatory cytokines in acute lung injury through the MyD88/NF-kappaB signaling pathway by targeting TLR4. Exp. Ther. Med. 16, 3913–3920 30344669 10.3892/etm.2018.6692 PMC6176196

[19] Zhang Q., Weng Y., Jiang Y., Zhao S., Zhou D. and Xu N. (2018) Overexpression of miR-140-5p inhibits lipopolysaccharide-induced human intervertebral disc inflammation and degeneration by downregulating toll-like receptor 4. Oncol. Rep. 40, 793–802 29901170 10.3892/or.2018.6488

[20] Li H., Guan S.B., Lu Y. and Wang F. (2017) MiR-140-5p inhibits synovial fibroblasts proliferation and inflammatory cytokines secretion through targeting TLR4. Biomed. Pharmacother. 96, 208–214 28987944 10.1016/j.biopha.2017.09.079

[21] Tu Z., Li Y., Dai Y., Li L., Lv G., Chen I. et al. (2017) MiR-140/BDNF axis regulates normal human astrocyte proliferation and LPS-induced IL-6 and TNF-alpha secretion. Biomed. Pharmacother. 91, 899–905 28501777 10.1016/j.biopha.2017.05.016

[22] Huang Y.F., Zhang Y., Liu C.X., Huang J. and Ding G.H. (2016) microRNA-125b contributes to high glucose-induced reactive oxygen species generation and apoptosis in HK-2 renal tubular epithelial cells by targeting angiotensin-converting enzyme 2. Eur. Rev. Med. Pharmacol. Sci. 20, 4055–4062 27775793

[23] Brownlee M. (2005) The pathobiology of diabetic complications: a unifying mechanism. Diabetes 54, 1615–1625 10.2337/diabetes.54.6.1615 15919781

[24] Taneda S., Honda K., Tomidokoro K., Uto K., Nitta K. and Oda H. (2010) Eicosapentaenoic acid restores diabetic tubular injury through regulating oxidative stress and mitochondrial apoptosis. American J. Physiol. Renal Physiol. 299, F1451–F1461 10.1152/ajprenal.00637.2009 20844021

[25] Sarniak A., Lipinska J., Tytman K. and Lipinska S. (2016) Endogenous mechanisms of reactive oxygen species (ROS) generation. Postepy Hig. Med. Dosw. 70, 1150–1165 10.5604/17322693.1224259 27892899

[26] Ni W.J., Tang L.Q. and Wei W. (2015) Research progress in signalling pathway in diabetic nephropathy. Diabetes Metab. Res. Rev. 31, 221–233 10.1002/dmrr.2568 24898554

[27] Yao L., Li J., Li L., Li X., Zhang R., Zhang Y. et al. (2019) Coreopsis tinctoria Nutt ameliorates high glucose-induced renal fibrosis and inflammation via the TGF-beta1/SMADS/AMPK/NF-kappaB pathways. BMC Complement. Alternat. Med. 19, 14 10.1186/s12906-018-2410-7 30630477 PMC6327481

[28] Chen F., Zhu X., Sun Z. and Ma Y. (2018) Astilbin inhibits high glucose-induced inflammation and extracellular matrix accumulation by suppressing the TLR4/MyD88/NF-kappaB pathway in rat glomerular mesangial cells. Front. Pharmacol. 9, 1187 10.3389/fphar.2018.01187 30459606 PMC6232904

[29] Rovira-Llopis S., Escribano-Lopez I., Diaz-Morales N., Iannantuoni F., Lopez-Domenech S., Andujar I. et al. (2018) Downregulation of miR-31 in diabetic nephropathy and its relationship with inflammation. Cell. Physiol. Biochem. 50, 1005–1014 10.1159/000494485 30355913

[30] Liu X.D., Zhang L.Y., Zhu T.C., Zhang R.F., Wang S.L. and Bao Y. (2015) Overexpression of miR-34c inhibits high glucose-induced apoptosis in podocytes by targeting Notch signaling pathways. Int. J. Clin. Exp. Pathol. 8, 4525–4534 26191142 PMC4503014

[31] Wang G., Yan Y., Xu N., Hui Y. and Yin D. (2019) Upregulation of microRNA-424 relieved diabetic nephropathy by targeting Rictor through mTOR complex 2/protein kinase B signaling. J. Cell. Physiol. 234, 11646–11653 10.1002/jcp.27822 30637733

[32] Yu F.N., Hu M.L., Wang X.F., Li X.P., Zhang B.H., Lu X.Q. et al. (2019) Effects of microRNA-370 on mesangial cell proliferation and extracellular matrix accumulation by binding to canopy 1 in a rat model of diabetic nephropathy. J. Cell. Physiol. 234, 6898–6907 10.1002/jcp.27448 30317577

[33] Yao T., Zha D., Gao P., Shui H. and Wu X. (2018) MiR-874 alleviates renal injury and inflammatory response in diabetic nephropathy through targeting toll-like receptor-4. J. Cell. Physiol. 234, 871–879 10.1002/jcp.26908 30171701

[34] Wagener F.A., Dekker D., Berden J.H., Scharstuhl A. and van der Vlag J. (2009) The role of reactive oxygen species in apoptosis of the diabetic kidney. Apoptosis 14, 1451–1458 10.1007/s10495-009-0359-1 19466552 PMC2773115

[35] Donate-Correa J., Martin-Nunez E., Muros-de-Fuentes M., Mora-Fernandez C. and Navarro-Gonzalez J.F. (2015) Inflammatory cytokines in diabetic nephropathy. J. Diabetes Res. 2015, 948417 10.1155/2015/948417 25785280 PMC4345080

[36] Liu Y. (2011) Cellular and molecular mechanisms of renal fibrosis. Nat. Rev. Nephrol. 7, 684–696 10.1038/nrneph.2011.149 22009250 PMC4520424

[37] Verzola D., Bertolotto M.B., Villaggio B., Ottonello L., Dallegri F., Frumento G. et al. (2002) Taurine prevents apoptosis induced by high ambient glucose in human tubule renal cells. J. Investig. Med. 50, 443–451 10.1136/jim-50-06-04 12425431

[38] Yang Z., Guo Z., Dong J., Sheng S., Wang Y., Yu L. et al. (2018) miR-374a regulates inflammatory response in diabetic nephropathy by targeting MCP-1 expression. Front. Pharmacol. 9, 900 10.3389/fphar.2018.00900 30147653 PMC6095963

[39] Nagy B. Jr, Nagy B., Fila L., Clarke L.A., Gonczy F., Bede O. et al. (2016) Human epididymis protein 4: a novel serum inflammatory biomarker in cystic fibrosis. Chest 150, 661–672 10.1016/j.chest.2016.04.006 27105680

[40] Zhao L., Qi Y., Xu L., Tao X., Han X., Yin L. et al. (2018) MicroRNA-140-5p aggravates doxorubicin-induced cardiotoxicity by promoting myocardial oxidative stress via targeting Nrf2 and Sirt2. Redox Biol. 15, 284–296 10.1016/j.redox.2017.12.013 29304479 PMC5975069

[41] Liao W., Fu Z., Zou Y., Wen D., Ma H., Zhou F. et al. (2017) MicroRNA-140-5p attenuated oxidative stress in Cisplatin induced acute kidney injury by activating Nrf2/ARE pathway through a Keap1-independent mechanism. Exp. Cell Res. 360, 292–302 10.1016/j.yexcr.2017.09.019 28928081

[42] Yuan S., Liu X., Zhu X., Qu Z., Gong Z., Li J. et al. (2018) The role of TLR4 on PGC-1alpha-mediated oxidative stress in tubular cell in diabetic kidney disease. Oxid. Med. Cell. Longev. 2018, 6296802 10.1155/2018/6296802 29861832 PMC5976914

[43] Shen J., Liu L., Zhang F., Gu J. and Pan G. (2019) LncRNA TapSAKI promotes inflammation injury in HK-2 cells and urine derived sepsis-induced kidney injury. J. Pharm. Pharmacol. 71, 839–848 10.1111/jphp.13049 30666657

[44] Ye H.Y., Jin J., Jin L.W., Chen Y., Zhou Z.H. and Li Z.Y. (2017) Chlorogenic acid attenuates lipopolysaccharide-induced acute kidney injury by inhibiting TLR4/NF-kappaB signal pathway. Inflammation 40, 523–529 10.1007/s10753-016-0498-9 28028753

[45] Ma F., Li L., Wang Q., Chen Z., You Y., Gao P. et al. (2019) Qi-dan-di-huang decoction alleviates diabetic nephropathy by inhibiting the NF-kappaB pathway. Front. Biosci. 24, 1477–1486 10.2741/4792 31136992

[46] Wang H.Q., Wang S.S., Chiufai K., Wang Q. and Cheng X.L. (2019) Umbelliferone ameliorates renal function in diabetic nephropathy rats through regulating inflammation and TLR/NF-kappaB pathway. Chinese J. Nat. Med. 17, 346–354 10.1016/S1875-5364(19)30040-8 31171269

[47] Zhu L., Han J., Yuan R., Xue L. and Pang W. (2018) Berberine ameliorates diabetic nephropathy by inhibiting TLR4/NF-kappaB pathway. Biol. Res. 51, 9 10.1186/s40659-018-0157-8 29604956 PMC5878418

[48] Xie W., Santulli G., Reiken S.R., Yuan Q., Osborne B.W., Chen B.X. et al. (2015) Mitochondrial oxidative stress promotes atrial fibrillation. Sci. Rep. 5, 11427 10.1038/srep11427 26169582 PMC4501003

[49] Santulli G., Xie W., Reiken S.R. and Marks A.R. (2015) Mitochondrial calcium overload is a key determinant in heart failure. Proc. Natl. Acad. Sci. U.S.A. 112, 11389–11394 10.1073/pnas.1513047112 26217001 PMC4568687

[50] Lee H.B., Yu M.R., Yang Y., Jiang Z. and Ha H. (2003) Reactive oxygen species-regulated signaling pathways in diabetic nephropathy. J. Am. Soc. Nephrol. 14, S241–S245 10.1097/01.ASN.0000077410.66390.0F 12874439

[51] Nishikawa T. and Araki E. (2007) Impact of mitochondrial ROS production in the pathogenesis of diabetes mellitus and its complications. Antioxid. Redox Signal. 9, 343–353 17184177 10.1089/ars.2006.1458

[52] Kiritoshi S., Nishikawa T., Sonoda K., Kukidome D., Senokuchi T., Matsuo T. et al. (2003) Reactive oxygen species from mitochondria induce cyclooxygenase-2 gene expression in human mesangial cells: potential role in diabetic nephropathy. Diabetes 52, 2570–2577 10.2337/diabetes.52.10.2570 14514642

